# Emotions and behaviours of child and adolescent psychiatric patients during the COVID-19 pandemic

**DOI:** 10.1192/bjo.2023.533

**Published:** 2024-02-16

**Authors:** Claudine Laurent-Levinson, Anne-Sophie Pellen, Hugues Pellerin, Cyril Hanin, Juliette Bouzy, Marie Devernay, Vanessa Milhiet, Xavier Benarous, Angèle Consoli, Jianxin Shi, Douglas F. Levinson, David Cohen

**Affiliations:** Department of Child and Adolescent Psychiatry, Centre de Référence des Maladies Rares à Expression Psychiatrique, Pitié-Salpêtrière University Hospital, Assistance Publique-Hôpitaux de Paris, France; and Groupe de Recherche Clinique n 15 – Troubles Psychiatriques et Développement (PSYDEV), Faculty of Medicine, Sorbonne Université, Paris, France; Department of Child and Adolescent Psychiatry, Centre de Référence des Maladies Rares à Expression Psychiatrique, Pitié-Salpêtrière University Hospital, Assistance Publique-Hôpitaux de Paris, France; Unité de Médecine pour Adolescents, Armand-Trousseau Hospital, Assistance Publique-Hôpitaux de Paris, France; Groupe de Recherche Clinique n 15 – Troubles Psychiatriques et Développement (PSYDEV), Faculty of Medicine, Sorbonne Université, Paris, France; and Department of Child and Adolescent Psychiatry, Pitié-Salpêtrière University Hospital, Assistance Publique-Hôpitaux de Paris, France; Groupe de Recherche Clinique n 15 – Troubles Psychiatriques et Développement (PSYDEV), Faculté de Médecine Sorbonne Université, Paris, France; and Department of Child and Adolescent Psychopathology, Amiens University Hospital, Amiens, France; Biostatistics Branch, Division of Cancer Epidemiology and Genetics, National Cancer Institute, Bethesda, Maryland, USA; Groupe de Recherche Clinique n 15 – Troubles Psychiatriques et Développement (PSYDEV), Faculty of Medicine, Sorbonne Université, Paris, France; and Department of Psychiatry and Behavioral Sciences, Stanford University, Stanford, California, USA; Department of Child and Adolescent Psychiatry, Centre de Référence des Maladies Rares à Expression Psychiatrique, Pitié-Salpêtrière University Hospital, Assistance Publique-Hôpitaux de Paris, France; Groupe de Recherche Clinique n 15 – Troubles Psychiatriques et Développement (PSYDEV), Faculty of Medicine, Sorbonne Université, Paris, France; and CNRS UMR 7222, Institute for Intelligent Systems and Robotics, Sorbonne University, Paris, France

**Keywords:** Children and adolescents, psychiatric disorders, COVID-19, pandemic, resilience

## Abstract

**Background:**

Previous pandemics have had negative effects on mental health, but there are few data on children and adolescents who were receiving ongoing psychiatric treatment.

**Aims:**

To study changes in emotions and clinical state, and their predictors, during the COVID-19 pandemic in France.

**Method:**

We administered (by interview) the baseline Youth Self-Report version of the CoRonavIruS Health Impact Survey v0.3 (CRISIS, French translation) to 123 adolescent patients and the Parent/Caregiver version to evaluate 99 child patients before and during the first ‘lockdown’. For 139 of these patients who received ongoing treatment in our centre, treating physicians retrospectively completed longitudinal global ratings for five time periods, masked to CRISIS ratings.

**Results:**

The main outcome measure was the sum of eight mood state items, which formed a single factor in each age group. Overall, this score improved for each age group during the first lockdown. Clinician ratings modestly supported this result in patients without intellectual disability or autism spectrum disorder. Improvement of mood states was significantly associated with perceived improvement in family relationships in both age groups.

**Conclusions:**

Consistent with previous studies of clinical cohorts, our patients had diverse responses during the pandemic. Several factors may have contributed to the finding of improvement in some individuals during the first lockdown, including the degree of family support or conflict, stress reduction owing to isolation, limitations of the outcome measures and/or possible selection bias. Ongoing treatment may have had a protective effect. Clinically, during crises additional support may be needed by families who experience increased conflict or who care for children with intellectual disability.

The worldwide pandemic of coronavirus disease-2019 (COVID-19; SARS-CoV-2) required large-scale confinement (‘lockdown’) of populations starting in early 2020, resulting in disruptions of all aspects of life. Previous pandemics have been associated with harmful effects on mental health,^1^ particularly in children and parents.^2^ This motivated a large set of studies of population-based and clinical cohorts to evaluate these effects during the COVID-19 pandemic and to identify aggravating and mitigating factors. We initiated the present study to evaluate these issues in children and adolescents who were receiving psychiatric treatment in affiliated services of our department, using our French-language version of the CoRonavIruS Health Impact Survey (CRISIS)^3,4^ plus diagnostic and global clinical ratings.

Large population-based studies revealed increases in depression and/or anxiety in adults during the first lockdown,^5–7^ including those with previous mental health problems,^6,7^ with considerable within-study variability.^3,8–10^ Similar findings have been reported in most population-based studies of children and adolescents during versus before the first lockdown. For example: parents of ~40 000 Chicago children and adolescents reported worsening of all mental health-related variables;^11^ anxiety and depression scores were markedly increased in US females (*n* = 451, ages 12–22);^12^ parents of 1011 US children/adolescents reported worsening mental health in children (14.5%) and parents (27%), often in the same families;^13^ in a follow-up survey, 248 Australian adolescents reported increased depression and anxiety, associated with family conflict;^14^ parents reported a doubling of psychiatric disorders in 552 Bangladeshi children/adolescents;^15^ and parents of 105 US children in a longitudinal study reported marked increases in mental health symptoms.^16^

However, there was variability across and within studies of the first lockdown: longitudinal self-ratings of 322 mostly Hispanic US children (aged 10–14) showed improvement of externalising, internalising and attention problems in those with, and of internalising problems in those without, previous mental health problems;^17^ 2130 Australian parents reported both positive and negative family changes;^18^ and US emergency department visits for youth mental health problems declined^19^ (although this could also reflect reluctance to enter emergency treatment settings).

In children/adolescents who were in active psychiatric treatment when the pandemic started, studies have reported overall worsening, mixed or mostly positive changes^20,21^ (see Discussion for a review). We hypothesised that the present study would detect overall worsening, based on population-based studies during previous pandemics that reported on individuals with and without previous mental disorders, but not on those who were receiving active treatment during the crisis period.

Our objectives were to investigate whether children and adolescents who were receiving psychiatric treatment experienced significant changes in their emotional state during the first confinement (lockdown) period and to identify factors that were associated with these changes.

## Method

### Recruitment

**Fig. 1:**
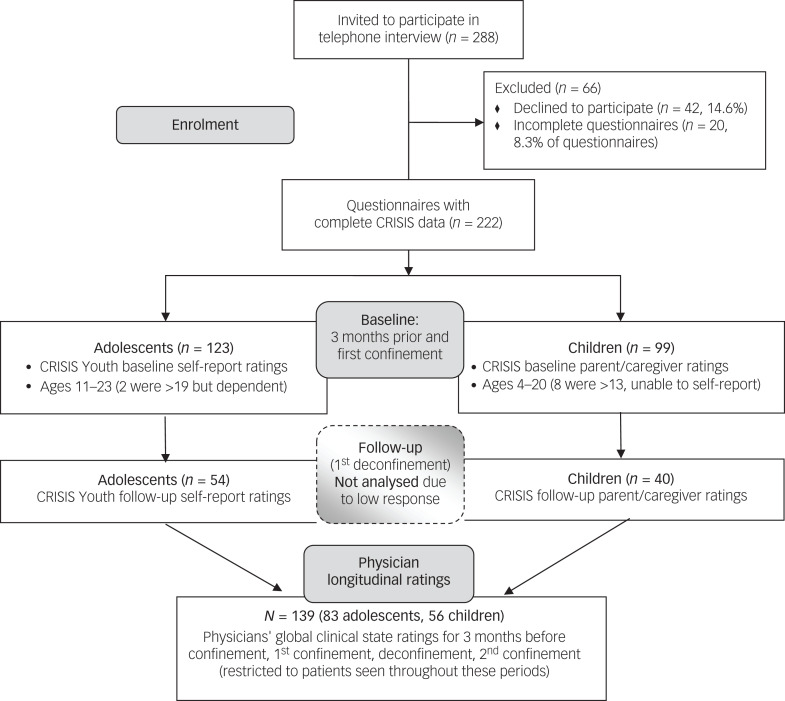
Flow diagram of recruitment and participation in the adolescent and child subcohorts. The COHORT diagram format (developed for clinical trials) is modified here to provide information about recruitment, inclusion, exclusion and main procedures in this observational study.

The first two confinement (lockdown) periods in France were 1 March to 11 May 2020 (CONF1) and 30 October to 15 December 2020 (CONF2). During CONF1, 33 clinicians (child psychiatrists, child psychiatry residents, behavioural paediatricians, psychologists) were asked to recruit their most recently seen patients from affiliated clinical services: out- and in-patient services of the Department of Child and Adolescent Psychiatry of Pitié-Salpêtrière University Hospital; a community child mental health programme (12th district of Paris); and an adolescent healthcare unit (Armand-Trousseau Hospital, Paris) (affiliates of Sorbonne University and Assistance Publique-Hôpitaux de Paris). Participants were patients who could self-rate (mostly adolescents) or parents of children (and some adolescents) who could not self-rate. See Fig. 1 for a flow diagram of enrolment and participation. We refer to these two cohorts as ‘adolescents’ (self-assessed) and ‘children’ (assessed by a parent or primary caregiver).

### CRISIS questionnaires

#### Description

The CRISIS questionnaires collect information about the effects of the COVID-19 pandemic.^4^ The main outcome measure is a set of mood state items related to depression and anxiety, considered a common pathway for distress based on the circumplex model of affect.^22^ There are versions for adults, youth (self-rated) and children (parent-rated), for baseline and follow-up periods, available online (along with translations, including our French versions^4^). We administered two baseline versions during CONF1: Youth (self-ratings by adolescents or older children) and Parent/Caregiver (‘parent’ ratings of children and more dependent adolescents).

Subsets of baseline items (rated only once, for the current 2 weeks) address background, COVID-19 illness/exposure and related life changes. Potential outcome items were rated for two separate time periods (the 3 months prior to the pandemic and the current 2 weeks) to assess mood states, daily behaviours, media use and substance use. Health, educational and recreational supports/resources are queried. We also administered follow-up Youth Self-Report and Parent/Caregiver versions during the first ‘deconfinement’ (lifting of lockdown), but excluded these data from analysis owing to poor response rate (see Administration section below).

#### Translation

We translated the Youth Self-Report and Parent/Caregiver baseline and follow-up questionnaires (versions 0.3)^4^ into French, with minor modifications reflecting French educational and healthcare systems. Initial drafts were generated with Google Translate, edited and revised by three clinicians, and back-translated by a bilingual individual, who proposed further revisions.

#### Administration

Baseline questionnaires were administered during CONF1. CRISIS was developed using online self-administration,^3^ but to maximise response and completion rates we administered it by telephone interview (or in-person for adolescent in-patients). Because clinicians were unusually busy, we recruited various trusted interviewers (child psychiatrists and residents, paediatricians, psychologists, research assistants, speech therapists, nurses, nursing assistants and medical secretaries). They were instructed to: (a) contact a parent of each patient to explain the study and obtain consent; and (b) read each CRISIS item verbatim to the participating patient or parent/caregiver. We attempted to reach participants during the first deconfinement for a follow-up interview. Responses were recorded on paper forms or (later) a web-based form.

#### Outcome variables

The original CRISIS study of large non-clinical cohorts reported that confirmatory factor analysis showed good fit of the 10 mood state items as one factor; outcome was the change in these items across time periods: worried, happy/sad, relaxed/anxious, fatigued, concentration, irritability, lonely, negative thoughts, enjoying activities, agitated,^3^ rated 1 to 5 (5 indicated worst in our analyses).

However, in our two smaller cohorts, fit statistics for the confirmatory factor analysis were unacceptable (ratings for the 3 months before confinement): the goodness-of-fit indices were 0.784 (adolescents) and 0.729 (children); the comparative fit indices were 0.887 and 0.782; the root mean square errors of approximations were 0.083 and 0.120; the root mean square residuals were 0.102 and 0.174. In exploratory principal components analyses, eight items (all except Enjoying Activities and Agitated) loaded on one factor at >0.39 in each cohort; a two-factor solution was difficult to interpret.

We therefore adopted the raw sum of these ‘Mood8’ items as the primary outcome scale. We computed ‘Mood8-Before’ (rated retrospectively for the 3 months prior to the pandemic), ‘Mood8-CONF1’ (rated for the 2-week period before the interview, during CONF1) and ‘Mood8-Change’ (Mood8-CONF1 minus Mood8-Before). Mood8 correlated highly with 10-item factor scores from principal components analyses (e.g. in adolescents: Before *r* = 0.921, During *r* = 0.938, Change *r* = 0.910), but raw scores were easier to compare across cohorts. Change in substance use was dropped as an outcome because usage rates were low (see Results).

#### Predictor variables

In each cohort, two types of variable were utilised in a multivariable analysis as predictors of Mood8-Change. These included the most common psychiatric diagnoses in each cohort (see Physicians’ ratings section below) and a set of CRISIS variables which were rated only once (for the current 2-week period during CONF1) to define aspects of the patient's household environment and the impact of COVID-19 on the household.

The 12 ‘life changes’ variables address potential pandemic-related predictors of distress: insecurity about food, housing or finances; difficulty following regulations; change in family relations, relations with friends or frequency of external conversations; stress about family changes, friendship changes, restrictions or event cancellations; and perception of positives during confinement. We performed principal components analyses on these variables within each subcohort, yielding solutions explaining 61.9% of variance (adolescents: five factors) or 58.2% (children: four factors) (Supplementary Tables 1 and 2, available at https://doi.org/10.1192/bjo.2023.533).

Other CRISIS predictors were: (a) COVID impact (sum of the variables: any family impact; family member diagnosed; 2-week exposure (of self); 2-week symptom count (for self)); (b) COVID worries (sum of the following variables: worried about self; worried about others; physical worries; mental worries; reading and talking about crisis; pessimism/optimism); (c) number of adults at home; (d) number of residents per room; (e) presence of an essential/front-line worker in the home; and (f) percentage of resources that were reduced or interrupted (the questionnaire inquired about pre-pandemic psychiatric, medical, educational and recreational resources and whether each was continued, reduced or interrupted during CONF1).

### Physicians’ ratings

Two sets of diagnostic and severity ratings were collected from the treating physicians (child psychiatrist or behavioural paediatrician).

#### Baseline ratings

The physician (on a paper form) rated presence/absence of 20 ICD-10 diagnoses (separately for before and during CONF1, or a single lifetime rating for attention-deficit hyperactivity disorder, autism spectrum disorder (ASD), intellectual disability and specific learning difficulties) allowing multiple diagnoses per patient (see Supplementary Tables 3 and 4). All analyses of CRISIS predictor variables treated each diagnosis as present (i.e. assigned by the physician during and/or prior to CONF1) or absent (never assigned). Additional ratings for CONF1 were Clinical Global Impressions-Severity (CGI-S)^23^ (1 = not at all ill; 7 = among the most extremely ill) and Global Assessment of Functioning (GAF)^24,25^ for age <15 or ≥15 years).

#### Longitudinal global ratings

Less than 50% of participants completed a follow-up questionnaire. Therefore, to seek additional longitudinal information and to complement findings from self-ratings, after CONF2 we asked physicians for retrospective ratings (using an online form, masked to CRISIS ratings) for each of five periods: Past (>3 months before CONF1); Before (the 3 months before CONF1); CONF1 (during CONF1); DECONF (during first deconfinement); CONF2 (during second confinement). These ratings included the 20 diagnoses listed above (present/absent)**,** CGI-S (as described above) and ‘clinically significant aggravation’ (data not analysed here). Note that the lifetime intellectual disability and ASD diagnoses used in longitudinal analyses here were congruent in physicians’ baseline and longitudinal ratings. Clinicians rated patients with whom they had contact throughout the five periods, excluding patients seen for brief consultations, emergency treatment or in-patient treatment while receiving other treatment elsewhere (it was not feasible to solicit ratings from external clinicians during lockdown).

### Ethical approval and informed consent

The authors assert that all procedures contributing to this work comply with the ethical standards of the relevant national and institutional committees on human experimentation and with the Helsinki Declaration of 1975, as revised in 2008. All procedures involving human subjects/patients were approved by the Comité d'Éthique de la Recherche (Research Ethics Committee), Sorbonne University (protocol CER-2020-29). In France, oral consent is permitted when a survey is the only intervention. By email or in-person, the interviewer provided written information about the study in advance to one parent/caregiver and to participating adolescents, and obtained oral consent (for over-18s) or assent (for minors) from each person who was interviewed by telephone, or written consent if interviewed in-person.

### Statistical analyses

The CRISIS data were analysed separately for adolescents and children because of differences in age and informant (self or parent). All variables were expressed as higher score indicating worse. Two types of factor analysis were utilised: (a) to test whether the 10 mood state items formed a single factor, goodness-of-fit statistics were computed with weighted least squares confirmatory factor analysis; and (b) exploratory factor analyses were carried out for each of two sets of variables (mood state and life changes items) using principal components analyses on correlation matrices, with Varimax rotation. For pairwise correlations, Pearson correlations are reported; Spearman rank-order correlations gave similar results.

To test the primary outcome in each cohort, linear mixed model (LMM) analyses of participants (random effect, intercept) and time (fixed effect) were carried out to evaluate whether Mood8 scores improved or worsened during CONF1 compared with the 3 months before. Age and gender (fixed effects) were then added as covariates to test the robustness of any time effect (Supplementary Table 8). Models were fitted by restricted maximum likelihood using R package *lme4.*^26^ Possible covariate effects on Mood8-Change scores were also examined with Pearson correlations between age and Mood8-Change and *t*-tests of Mood8-Change in males versus females (Supplementary Table 7).

Then, multivariable analyses were performed to determine whether selected CRISIS variables and common diagnoses (present during or before CONF1 versus absent) were significantly associated with changes in Mood8 scores. For each cohort, multiple linear regression of predictor variables was performed (in R, version 4.2.1 for Windows) on Mood8-Change scores (‘full model’), followed by LASSO (least absolute shrinkage and selection operator) regression analysis of the same model (R package *glmnet*^27^) to identify significant predictor variables (those with non-zero LASSO beta values). The tuning parameter λ was chosen based on the λ–CVM (cross-validated mean error) curve generated using five-fold cross-validation. Finally the linear model was refitted using the selected variables (‘reduced model’). There were 16 predictor variables for adolescents and 14 for children, because the cohorts differed in having five versus four life changes factors and 6 versus 5 common diagnoses (in adolescents: any anxiety disorder, major depression, ADHD, ASD and intellectual disability; in children: conduct disorder replaced depression and ASD was omitted because 19 of the 22 children with ASD also had intellectual disability). To aid in the interpretation of associations of Mood8-Change with life changes factor scores, Pearson correlations were computed between Mood8-Change score and the individual variables that loaded on each associated factor score.

Finally, exploratory LMM analyses were performed with *lme4* for 139 children and adolescents with clinician CGI-S ratings across five time periods, to determine whether they also demonstrated improvement during CONF1 and to explore change after CONF1. We also tested the interaction between time and diagnosis (presence/absence of intellectual disability and/or ASD) because (a) most of the participants with intellectual disability were children in whom intellectual disability and ASD largely overlapped and (b) Mood8 changed little in participants with intellectual disability in both cohorts during CONF1 (Table 3; Supplementary Tables 5 and 6). The full model included the following variables: participant (random effect), time (fixed), diagnosis (fixed) and time × diagnosis (fixed). For each relevant effect (Supplementary Table 9), a likelihood ratio test was performed (after maximising likelihoods with *lme4*) to contrast one LMM that included and one that omitted the test effect(s).

For example, time (fixed effect) was parameterised as four dummy variables (time 1 as baseline; dummy variables for times 2–5). The likelihood ratio for time compared an LMM for participant and time with one for participant. The resulting statistic follows 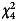 under the null hypothesis of no change across time. (Linear trend was not tested, because monotonic change was not expected during alternating periods with and without lockdown.) The time × diagnosis interaction was tested by comparing the global model with a model that omitted the interaction (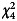 under the null hypothesis of no interaction).

Then, because time, diagnosis and time × diagnosis were significant (Results section and Supplementary Table 9), *post hoc* analysis was performed for each pair of adjacent periods in the subgroup with no intellectual disability/ASD. LMM models included participant (random effect, intercept) and time (fixed effect with one dummy variable for the pair of adjacent periods) with model fitting by restricted maximum likelihood and Wald tests of statistical significance.

Analyses were performed with SYSTAT 13.0 for Windows (Systat Software Inc., San Jose, California, USA; see https://systatsoftware.com/) (for *t*-tests, factor analyses and correlations) and R packages (linear, LASSO and LMM regression) as noted above.

## Results

### Demographic and clinical characteristics

Figure 1 describes recruitment and participation in the two subcohorts. Of 288 invited to participate, 242 (84%) were interviewed, with 20 (8.3%) incomplete and 222 complete questionnaires (*n* = 123 in the self-rated adolescent cohort and *n* = 99 in the parent-rated child cohort). Table 1 provides demographic information on these cohorts and on the subset (*n* = 139) with longitudinal clinician ratings. Age ranges were 11–23 years for adolescents (two were >19 but highly dependent and living at home) and 4–20 years for children (eight were >13 but unable to self-rate).

**Table 1:** Characteristics of the cohorts

	Adolescent^a^ (self-rated)	Child^a^ (parent-rated)	Longitudinal^a^ (psychiatrist-rated, both age groups)
Participants, *n*	123	99	139
Female, *n* (%)	64 (52.0%)	70 (70.1%)	74 (53.2%)
Male, *n* (%)	55 (44.7%)	28 (28.3%)	60 (43.2%)
Transgender or non-binary, *n* (%)	4 (3.3%)	1 (1%)	5 (3.6%)
(Adolescent cohort), *n* (%)			83 (59.7%)
(Child cohort), *n* (%)			56 (40.3%)
Age, years: mean (s.d.)	15.1 (2.00)	9.8 (3.01)	13.0 (3.66)
Age range, years	11–23	4–20	4–23
Most common diagnoses, *n* (%)^b^			
Any anxiety disorder	65 (52.8%)	53 (53.5%)	84 (60.4%)
Major depression	44 (35.8%)	10 (10.1%)	32 (23.0%)
Conduct disorder	17 (10.6%)	22 (22.2%)	28 (20.1%)
Eating disorder	13 (10.6%)	6 (6.1%)	18 (12.9%)
ADHD	24 (19.5%)	34 (34.3%)	35 (25.2%)
Intellectual disability	7 (5.7%)	30 (30.3%)	26 (18.7%)
ASD	10 (8.1%)	21 (21.2%)	26 (18.7%)

ADHD, attention-deficit hyperactivity disorder; ASD, autism spectrum disorder.

a. The adolescent (self-rated) group included 2 patients over age 19 who lived with family in a manner typical of adolescents; the child (parent-rated) group included 8 patients over age 13 who were not capable of self-rating. The longitudinal group included the 83 adolescent and 56 child participants for whom the treating psychiatrist could confidently assign Clinical Global Impressions-Severity ratings for the five time periods (>3 months before the first confinement; the 3 months before the first confinement; during the first confinement; during the first deconfinement; during the second confinement).

b. Frequencies of the most common ICD-10 clinical diagnoses are shown (individuals could receive more than one diagnosis). Frequencies of all diagnoses are shown in Supplementary Table 3 (adolescent group) and Supplementary Table 4 (child group).

Diagnosis frequencies (permitting multiple diagnoses per patient) are shown in Supplementary Tables 3 and 4. Most frequent were: among the adolescents – any anxiety disorder (52.8%), major depressive disorder (44%), ADHD (19.5%) and specific learning difficulties (29.3%, comorbid); among the children – any anxiety disorder (53.5%), ADHD (34.3%), intellectual disability (30.3%), conduct disorder (22%), ASD (21% – 19/22 also had intellectual disability) and specific learning difficulties (33.3%).

### Change in Mood8

**Fig. 2:**
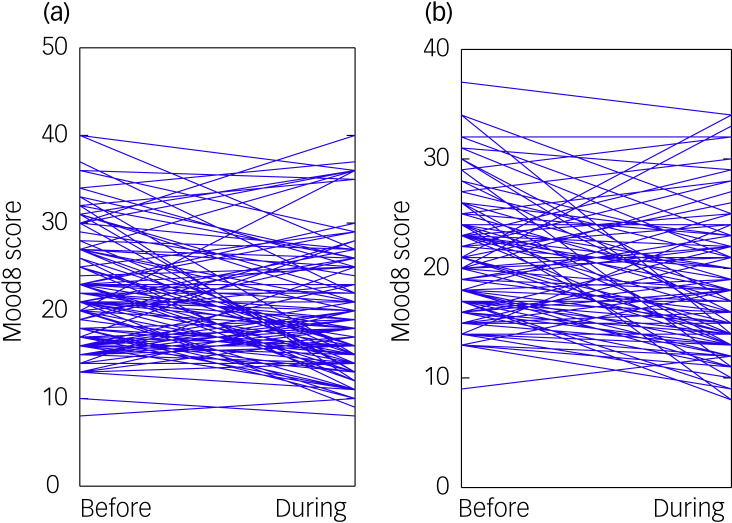
Mood8 scores before and during the first confinement for (a) 123 adolescent participants and (b) 99 child participants receiving psychiatric treatment. Each line shows the Mood8 score of one participant during the 3 months before (to the left) and during the first confinement (to the right).

In each cohort, LMM analyses showed that Mood8 scores significantly declined (improved) during CONF1 compared with the 3 months before confinement (Fig. 2), with significant effects of time in the adolescent cohort (MOOD8-Before: mean 21.894, s.d. = 6.60; Mood8-CONF1: mean 19.69, s.d. = 6.96; *t* = −3.672, d.f. = 122, *P* = 0.00036) and in the child cohort (Mood8-Before: mean 20.77, s.d. = 5.68; Mood8-CONF1: mean 18.43, s.d. = 6.18; *t* = −3.765, d.f. = 98, *P* = 0.00028). The time effect remained robust in each cohort after adjusting for age and for age and gender (Supplementary Table 8). Mood8 scores were higher in older than in younger adolescents (Supplementary Table 8), but Mood8-Change was unrelated to age or gender (Supplementary Table 7).

To estimate the proportions of participants experiencing clinically relevant change, we adopted a cut-off of 1 s.d. of change (s.d. = 6.65 in adolescents and s.d. = 6.617 in children: 29 adolescents (23.6%) improved and 10 worsened (8.1%) by ≥1 s.d. unit; 22 children improved (22.2%) and 5 worsened (5.1%)).

### Predictors and correlates of MOOD8 scores and changes

The results of multivariable analyses of predictors of Mood8-Change scores are shown in Table 2 (adolescents) and Table 3 (children). In both cohorts, variance inflation factors (VIF) were low, indicating low multicollinearity. Shown are the *P*-values of all predictor variables from multiple linear regression and then the beta values from LASSO regression, which selects variables with non-zero betas as a sparse model of significant predictors. In the lower part of each table, results of linear regression are shown for the reduced model (variables selected by LASSO regression). For adolescents and children respectively, optimal lambda values for Lasso regression were 1.08 and 1.98. Lasso selected four variables (adolescents) or one variable (children) with significant association with Mood8-Change (indicated by non-zero Lasso beta values). Adjusted squared multiple R values were 0.350 (full model) and 0.307 (reduced model) for adolescents and 0.319 and 0.184 for children.

**Table 2:** Association of Mood8 change scores with CRISIS variables and common diagnoses in the adolescent cohort (*n* = 123)

	Linear regression				LASSO beta
Variable	VIF	Beta	s.e.	*P*	
	Full linear regression model				
Life changes factor 1	1.14	0.0615	0.5590	0.913	
Life changes factor 2	1.16	1.8378	0.5659	**0.0016**	1.1384
Life changes factor 3	1.11	1.9173	0.5515	**0**.**0007**	0.8909
Life changes factor 4	1.04	0.1586	0.5348	0.767	
Life changes factor 5	1.08	0.1262	0.5456	0.818	
COVID impact	1.15	0.0067	0.2861	0.981	
COVID worries	1.21	0.3652	0.1530	**0**.**019**	0.1265
Essential worker in home	1.14	0.5951	1.2844	0.644	
Percentage of resources reduced/interrupted	1.16	1.3614	1.7000	0.425	
Number of adults in home	1.22	−0.3973	0.9037	0.661	
Number of residents per room	1.30	0.3830	1.6984	0.822	
Any anxiety disorder	1.16	−0.1502	1.1297	0.895	
Major depression	1.13	−3.5632	1.1572	**0**.**0026**	−0.8263
ADHD	1.10	1.7866	1.3815	0.199	
ASD	1.22	−3.8391	2.1120	0.072	
Intellectual disability	1.25	2.4409	2.7065	0.369	
**Reduced model (variables selected by LASSO)**					
Life changes factor 2	1.02	2.0486	0.5140	**0**.**0001**	
Life changes factor 3	1.08	1.9615	0.5305	**0**.**0003**	
COVID worries	1.07	0.3680	0.1398	**0**.**0096**	
Major depression	1.04	−3.4561	1.0818	**0**.**0018**	

VIF, variable inflation factor; LASSO, least absolute shrinkage and selection operator; ADHD, attention-deficit hyperactivity disorder; ASD, autism spectrum disorder; bold text indicates *p* < 0.05.

**Table 3:** Association of Mood8 change scores with CRISIS variables and common diagnoses in the child cohort (*n* = 99)

	Linear regression				LASSO beta
Variables	VIF	Beta	s.e.	*P*	
	Full linear regression model				
Life changes factor 1	1.12	2.4929	0.5867	**0.00006**	0.65
Life changes factor 2	1.30	0.5844	0.6326	0.358	
Life changes factor 3	1.30	0.2069	0.6321	0.744	
Life changes factor 4	1.12	0.0631	0.5868	0.915	
COVID impact	1.18	−0.0184	0.3314	0.956	
COVID worries	1.42	0.2162	0.1543	0.165	
Essential worker in home	1.14	2.3124	1.4972	0.126	
Percentage of resources reduced/interrupted	1.05	−0.3508	1.8922	0.853	
Number of adults in home	1.22	−1.0622	0.8671	0.224	
Number of residents per room	1.44	2.8547	1.2920	0.030	
Any anxiety disorder	1.76	1.2717	1.4709	0.390	
Conduct disorder	1.15	−0.4384	1.4250	0.759	
ADHD	1.32	2.0273	1.3259	0.130	
Intellectual disability	1.66	2.7853	1.5333	0.073	
**Reduced model (variable selected by LASSO)**					
Life changes factor 1		2.6477	0.5654	**0**.**000009**	

VIF, variable inflation factor; LASSO, least absolute shrinkage and selection operator; ADHD, attention-deficit hyperactivity disorder; ASD, autism spectrum disorder; bold text indicates *p* < 0.05.

For each cohort, the same variable(s) were selected as significant by linear regression and LASSO regression. In the adolescents, high scores on ‘life changes factor 1’ and ‘life changes factor 2’ and high ‘COVID worries’ ratings predicted worsening of Mood8 scores during CONF1, and a diagnosis of major depressive disorder predicted more improvement. In children, the ‘life changes factor 1’ score was associated with Mood8-Change and there was a trend toward participants with intellectual disability showing less improvement.

**Fig. 3:**
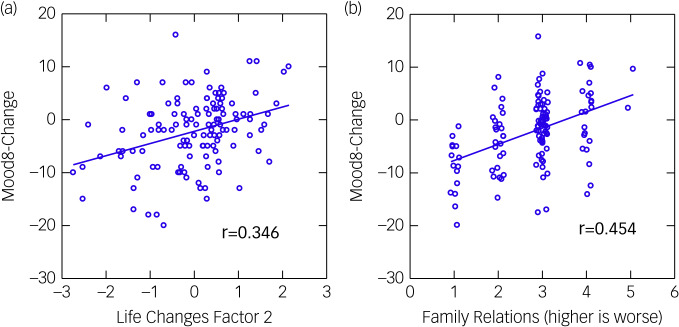
MOOD8-Change scores (Mood8-CONF1 minus Mood8-Before) plotted against (a) ‘life changes factor 2’ scores and (b) change in family relations. CONF1, the first confinement/lockdown.

The factor structure of the life changes items differed in the two cohorts (Supplementary Tables 1 and 2). To clarify the multiple regression results, Table 4 shows, for each cohort, the correlation between Mood8-Change and each individual questionnaire item that loaded highly (>0.4) on a life changes factor that was associated with Mood8-Change in multivariable analysis. The only item that was strongly correlated with Mood8-Change in both cohorts was change in family relations (‘During the past two weeks [during CONF1], has the quality of the relationships between you and members of your family changed?’, with ratings ranging from ‘A lot worse’ to ‘A lot better’; scores on this item were inverted for analysis so that 5 indicated ‘A lot worse’). Perceived improvement in family relations during CONF1 was associated with improvement in Mood8 scores in both cohorts, as illustrated for the adolescent cohort in Fig. 3. In the children, the other strongly significant association was with ‘positives’ (‘Has the COVID-19 crisis in your area led to any positive changes in your life?’, rated as ‘None,’ ‘Only a few’ or ‘Some’; we inverted scores on this item for analysis so that 3 = None.) The correlation shows that a perception of more positives during CONF1 was associated with improvement in Mood8 scores (and in family relationships).

**Table 4:** Correlations of Mood8-Change scores with individual ‘life changes’ items

	Adolescents		Children	
Items	*r*	*P*	*r*	*P*
Change in family relations (rev.)	**0.454**	**0**.**00000013**	**0**.**399**	**0**.**000042**
Difficulty following regulations	−0.108	0.233		
Family change stress	0.243	0.0067		
Friendship change stress	0.218	0.0153		
COVID restriction stress	0.228	0.0112		
Positives during CONF1 (rev.)			**0**.**425**	**0**.**000011**
Friendship changes (rev.)			0.142	0.161

rev., item reversed so that a high score indicated increased stress/difficulty; CONF1, the first confinement/lockdown. Pearson correlations and associated *P*-values are shown; bold text indicates *p* < 0.05.

Supplementary Tables 5 and 6 provide results of univariate analyses of Mood8 scores and changes in each cohort in participants with and without each common diagnosis. Numbers of participants with individual diagnoses were generally too low to provide clear information about differences among them in Mood8 scores and changes. Consistent with multivariable analyses, major depression was associated with greater improvement during CONF1 in adolescents, and intellectual disability with less improvement in children (in whom intellectual disability largely overlapped with ASD).

Substance misuse was not analysed because it was uncommon; for example, in the adolescents during CONF1, ‘use at least several times/month’ decreased from 11.4 to 2.4% (alcohol) and 7.3 to 0.8% (cannabis) and ‘use at least several times/week’ fell from 1.6 to 0% (alcohol) and 3.3 to 0.8% (cannabis).

### Longitudinal clinician ratings

There were 139 participants with clinician CGI-S ratings across the five time periods, including 83 of the 123 adolescents and 56 of the 99 children. We first confirmed that the results of the main analyses of Mood8-Change in these 139 were representative of the two full cohorts: Mood8 improved during CONF1 (Before: mean 21.38, s.d. = 6.42; CONF1: mean 19.54, s.d. = 6.76, paired *t* = −3.38, d.f. = 138, *P* = 0.0009); and Mood8-Change correlated strongly with change in family relationships (*r* = 0.46, *P* < 0.000001).

**Fig. 4:**
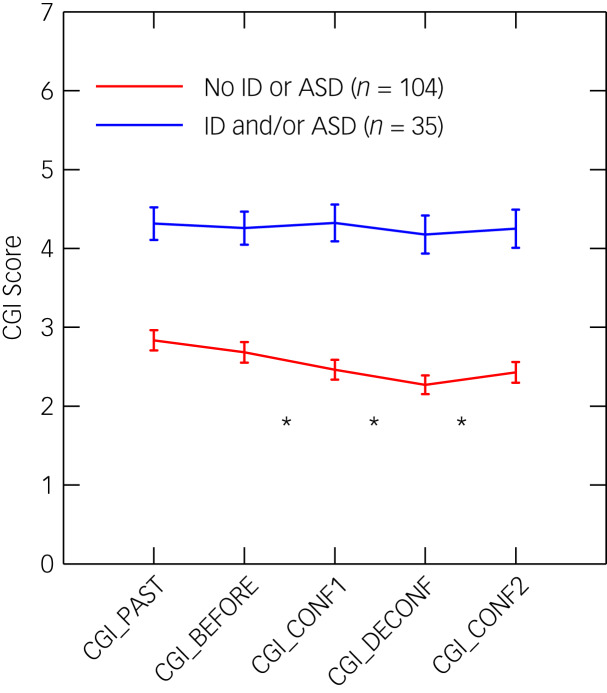
Clinician-rated Clinical Global Impressions-Severity (CGI-S) scores before and during two confinements for participants with (*n* = 35) and without (*n* = 104) a diagnosis of intellectual disability/autism spectrum disorder. Results are shown as the means (s.d.) of CGI-S scores across five time periods: Past (>3 months before CONF1, the first confinement/lockdown); Before (the 3 months before CONF1); during CONF1; during DECONF (first deconfinement); during CONF2 (second confinement). Asterisks mark the intervals with significant *post hoc* pairwise differences between adjacent time periods.

Figure 4 illustrates CGI-S scores across the five time periods for the 35 participants with diagnoses of intellectual disability and/or ASD and for the 104 participants without either diagnosis (see Method section). Details are shown in Supplementary Table 9. LMM analysis of time, diagnosis and the time × diagnosis interaction demonstrated significant effects for the entire model (χ^2^ = 101.94, d.f. = 9; *P* = 6.38 × 10^−18^), and for time (χ^2^ = 42.99, d.f. = 4; *P* = 1.04 × 10^−8^), diagnosis (χ^2^ = 31.24, d.f. = 1; *P* = 2.27 × 10^−8^) and the interaction (χ^2^ = 11.05, d.f. = 4; *P* = 0.026). Time (i.e. change across five time points) was found in *post hoc* tests to be significant in participants without intellectual disability or ASD (χ^2^ = 45.45, d.f. = 4; *P* = 3.20 × 10^−9^), but not in those with either diagnosis (χ^2^ = 2.63, *P* = 0.62).

For participants without intellectual disability or ASD, *post hoc* LMM pairwise tests for adjacent periods (Fig. 4) detected: improvement during CONF1 versus the 3 months before (beta = −0.146; s.e. = 0.069; *t* = −2.132, *P* = 0.033), which is consistent with the finding of improvement in Mood8 scores during CONF1; further improvement during DECONF versus CONF1 (beta = −0.154; s.e. = 0.069; *t* = −2.220; *P* = 0.026); and worsening (back to the CONF1 level) during CONF2 versus DECONF (beta = 0.149; s.e. = 0.071; *t* = 2.103; *P* = 0.035).

Mood8 scores were weakly correlated with clinicians’ CGI-S ratings, suggesting that they reflect partially overlapping clinical features: CGI-S *v.* Mood8 (Before, *r* = 0.166, *n* = 139, *P* = 0.0505; and CONF1, *r* = 0.216, *n* = 138, *P* = 0.011); and CGI-S *v.* Mood8-Change (*r* = 0.154, *n* = 138, *P* = 0.07).

## Discussion

During the first COVID-19 confinement, we administered the CRISIS questionnaire to 123 patients (mostly adolescents) and to a parent or caregiver of 99 patients (mostly children). There were two significant findings that were consistent across the two cohorts:

- on average, Mood8 scores (mood/worries items) improved during the first confinement
- changes in Mood8 scores were associated with perceived improvement or worsening of family relationships during confinement.

In the adolescent cohort, more worries about COVID-19 were also associated with worsening of Mood8 scores, and a diagnosis of major depressive disorder predicted greater improvement in Mood8 scores during CONF1.

The finding of significant improvement in Mood8 scores during CONF1 received some support from exploratory analyses of clinicians’ global severity ratings (CGI-S) that suggested improvement during CONF1 and DECONF, and worsening during CONF2 back to CONF1 levels.

Explanations for the main finding might include several factors:

- Self-selection bias can be a factor, especially in small cohorts. The cooperation rate (84%) argues against a major bias. However, clinicians were asked to propose the study to their most recently seen patients. Patients who had more regular therapeutic contact might have had advantageous characteristics (e.g. more involved and supportive families, better response to previous treatment, better relationships with clinicians). But our clinical experience was that patients and families who were having difficulties were eager for contact during confinement.
- Stress reduction: the first lockdown may have reduced daily stress from school and social interactions for some patients – as reported to interviewers by several participants. But this effect should be strongest in patients with anxiety disorders, whose Mood8-Change scores did not differ from those without anxiety disorders (Supplementary Tables 5 and 6).
- Limitations of the outcome measure: the CRISIS authors proposed that mood state ratings reflected a common pathway for crisis-related distress. But in retrospect, scales for externalising features would have been useful. Our physicians’ longitudinal severity ratings provide some support for improvement during the first confinement. There may also have been a ceiling effect: Fig. 2 shows that several adolescents had maximal scores before the lockdown, although a ceiling effect does not explain why all of them improved.
- Ongoing psychiatric treatment might have had a protective effect. As discussed in the introductory paragraphs and the literature review below, many population-based studies suggest that adults, children and adolescents with previous mental health problems are more psychologically vulnerable to pandemic-related stress. But those studies did not analyse subgroups currently receiving treatment. A protective effect could result from: previous stabilisation by medication and/or psychological treatments; ongoing medication; and ongoing psychological support and treatment for both the patient and parents. The clinicians rated global severity of illness as highest in the past (more than 3 months before the pandemic, which would include any initial acute period). Ongoing positive treatment effects might interact with other stabilising factors: for example, patients and families with a positive treatment experience might be more likely to remain in treatment and to be sufficiently stabilised to provide mutual support during a crisis. This might also have been reflected in the ratings of improved family relationships during confinement, which was associated with clinical improvement.

### Family relationships as a protective factor

Perceived improvement or worsening of family relationships during lockdown was strongly associated with change in Mood8 scores. We lack data to determine the directionality of this association – were patients partially protected from worsening if their families were able to create a supportive environment and/or did the family environment worsen because the patient was having more difficulty? Perhaps both, but as clinicians we suspect that family strengths and difficulties in the face of a crisis played a major role in mediating the degree of resilience or vulnerability of some of our patients.

### Our findings in the light of previous research

In children and adolescents, neither population-based nor clinical cohorts have shown exclusively negative results during the first COVID-19 lockdown. Population-based studies were reviewed in the introductory paragraphs. Some reports of clinical cohorts focused on negative outcomes: (a) parents of 441 Spanish psychiatric out-patients (≤18) rated more symptoms as worsened than as improved, with worsening associated with ASD and conduct disorders and with loss of income and parental stress;^28^ (b) parents’ CRISIS ratings showed worsening of loneliness, fatigue and lacking enjoyment in 111 German patients (ages 2–17, predominantly with ADHD, ASD, eating and tic disorders) and of worry and sadness in 173 controls;^29^ (c) parents rated 1013 Canadian children (2–18 years of age), including 594 clinical cases – worsening was reported in 70.2% in at least one domain (depression, anxiety, irritability, attention, hyperactivity and obsessions/compulsions), more so for the clinical group, and only 19.5% improved;^20^ (d) worsening in multiple domains was reported in 118 US individuals with ADHD and 120 controls (ages 15–17) (the only clinical cohort study with prospective pre-pandemic data);^30^ (e) parent ratings of 118 children with ASD (median age 6 years) showed increased behavioural problems in 44.9%, associated with family disruption (COVID-19 hospital admission or parental mental health problems);^31^ (f) according to parents’ online ratings, 371 UK children (with ADHD and/or ASD) were clinically worse compared with ‘published norms’ and a previous cohort;^32^ (g) parents rated 67% of 238 Italian children and adolescents with Tourette syndrome as worsening (tics, hyperactivity, rage, anxiety, obsessive–compulsive disorder).^33^

However, other reports documented a diversity of clinical changes during lockdown: (a) based on clinicians’ CGI ratings of 354 French clinical cases (ages 3–18), 40–55% were stable, 23–33% improved and 22–30% worsened;^21^ (b) equal proportions of 166 Tunisian patients (ages 2–20) were judged worse, stable or improved (patients with ADHD showed more improvement);^34^ (c) based on clinicians’ judgements of open-ended responses, parents described 533 children with ADHD (mean age 10.5; recruited online) as behaviourally worse (34.71%), stable (34.33%) or improved (30.96%);^35^ (d) parents’ CRISIS ratings of 213 children with ADHD (5–17 years of age) showed worsening of mood, enjoyment and loneliness, but 64% reported positive changes (e.g. more family time);^36^ (e) parent ratings of 37 Spanish children with ASD (aged 3–17) showed no clinical change.^37^

Thus, our findings are consistent with many previous population-based and clinical studies of children and adolescents during the COVID-19 pandemic: improvement in a substantial proportion of patients (perhaps with less worsening in our cohort than in most); a diversity of clinical responses; and a demonstrable impact of family factors in some studies.

### Limitations

We have noted the possibility of self-selection bias. We used retrospective baseline ratings (despite possible rater bias) because we lacked relevant previous data; only one clinical cohort study of children or adolescents could compare prospective pre-pandemic data with data collected during lockdown.^30^ Ratings of additional clinical domains would have been informative. Our results might not generalise to countries with different systems of healthcare and income support. Larger cohorts might detect effects of variables such as economic adversity. We lacked information about the duration of previous treatment as a possible predictor of response to the pandemic. Our French translation of the CRISIS interviews was not empirically validated prior to this study. Finally, we lacked data about the cumulative effects of multiple subsequent (and continuing) COVID-19 waves with additional morbidity and mortality, restrictions, economic distress, disconnection from school life and obstacles to the normal social, educational and recreational experiences of childhood. For example, in the UK, youth surveys suggest an increasing mental health burden starting in early 2021;^38^ and in France, government statistics showed that below age 15, emergency care for mood disorders increased late in 2020 and for suicidal behaviour during the first half of 2021 (compared with pre-COVID years).^39^

### Clinical implications

- For children and adolescents with and without ongoing psychiatric treatment, there are diverse psychological reactions to the pandemic. Some ‘rise to the occasion’ and do better than expected; others will have severe difficulties, especially over time. The overall trend in a given cohort (worsening, improvement) is less important than determining who is doing better versus worse, why and what can be done to help.
- For some patients and families, ongoing child psychiatric treatment might have been a protective factor. There have been massive efforts throughout the world to maintain ongoing child psychiatric treatment, while responding to those with new problems. Our results suggest that these efforts have been worthwhile.
- It may be particularly important to identify and support families who experience increased disorganisation and conflict during such a crisis.
- Some of our patients may have been relieved to stay home initially, but clinical experience suggests that some of these children then find the return to school quite stressful. Psychotherapeutic relationships are particularly important during these transitions.
- Most of our patients with intellectual disability were in the child cohort. They showed the least clinical change and the greatest global severity longitudinally. Most of these families live with chronically high stress in the face of their child's multiple challenges. During lockdown periods, most care arrangements were disrupted. This required a major re-adjustment by children and families. Our data suggest that they succeeded more often than might have been expected, but that they could benefit from more consistent support during future crises, particularly if family relations are deteriorating.

## Supporting information

Laurent-Levinson et al. supplementary material Laurent-Levinson et al. supplementary material

## Data Availability

Public access to the complete study dataset is not authorised for this study. Researchers who are interested in carrying out additional analyses of these data may contact the corresponding author, C.L.-L., to discuss obtaining an appropriately limited dataset for their purpose.
