# Supplementary material for: Emotions and behaviours of child and adolescent psychiatric patients during the COVID-19 pandemic
Source: BJPsych Open. 2024 Feb 16;10(2):e47. doi: 10.1192/bjo.2023.533 (PMC10897707; doi:10.1192/bjo.2023.533)
Supplement: Laurent-Levinson et al. supplementary material [file S2056472423005331sup001.pdf]

Supplementary Tables 1-9

Emotions and behaviors of child and adolescent psychiatric patients during the COVID-19 pandemic

C Laurent-Levinson et al.

Table S1: Life Changes factor analysis (adolescents)

Table S2: Life Changes factor analysis (children)

Table S3: Diagnoses of adolescent cases

Table S4: Diagnoses of child cases

Table S5: MOOD8 scores in relation to confinement and diagnosis (adolescents)

Table S6: MOOD8 scores in relation to confinement and diagnosis (children)

Table S7: Lack of association of Mood8-Change with Sex or Age

Table S8: Linear mixed model analysis of Mood8 scores during CONF1 vs. the three months before CONF1, with adjustment for age and sex

Table S9: Change in clinicians' CGI-Severity ratings (5 time periods, Linear Mixed Model regression): all subjects, and subgrouped by presence of ID and/or ASD

Table S1: Life Changes factor analysis (adolescents)

Variable	1 Insecurity	2 Family (rev)	3 Stress	4 Activities	5 Friends
Food insecurity	**0.727**	-0.026	-0.185	-0.103	-0.099
Housing insecurity	**0.708**	0.194	0.355	0.049	0.044
Financial difficulty	**0.705**	-0.226	0.332	0.134	0.107
Difficulty following regulations	-0.037	**-0.737**	0.284	-0.220	-0.080
**Change in family relations (rev)**	-0.097	**0.673**	0.342	-0.336	0.014
Stress about friendship changes	0.063	-0.013	***0.789***	-0.026	0.132
Stress about restrictions	0.225	-0.156	***0.681***	0.057	0.000
Stress about family changes	-0.016	***0.417***	***0.611***	0.123	0.085
Stress about Event Cancellation	0.041	-0.024	0.229	**0.833**	0.110
Change in relations with friends (rev)	0.075	-0.130	0.287	***-0.515***	**0.406**
Change in external conversations (rev)	-0.058	0.012	-0.100	-0.059	**0.804**
Positives during the crisis (rev)	0.043	0.094	0.211	0.083	**0.609**
**% of total variance explained**	*13.379*	*10.607*	*17.636*	*9.836*	*10.413*

Table S2: Life Changes factor analysis (children)

Variable	1 Relationships	2 Stress	3 Insecurity	4 External
**Change in family relations (rev)**	**0.806**	0.003	0.076	-0.222
Positives during the crisis (rev)	**0.582**	0.208	-0.115	0.061
Change in relations with friends (rev)	**0.510**	-0.034	**0.582**	0.213
Stress about Event Cancellation	-0.085	**0.683**	-0.030	0.048
Stress about family changes	0.384	**0.674**	0.106	-0.040
Stress about restrictions	0.252	**0.661**	-0.007	0.207
Stress about friendship changes	0.324	**0.640**	0.196	0.064
Food insecurity	-0.099	**0.639**	0.119	-0.259
Financial difficulty	0.008	0.006	**0.835**	-0.049
Housing insecurity	-0.302	**0.416**	**0.648**	-0.017
Change in external conversations (rev)	-0.096	-0.006	0.041	**0.907**
Difficulty following regulations	0.341	0.344	-0.073	0.286
**% tot variance explained**	*14.989*	*20.924*	*12.884*	*9.356*

Shown are the results of Principal Components Analysis of 12 Life Changes items in each cohort, after Varimax rotation, retaining factors with Eigenvalue>1.

Total variance explained is 61.9% in adolescents and 58.2% in children.

For individual items, "rev" indicates that the directionality of the rating was reversed so that a high rating would indicate changes that would typically be viewed as more adverse, e.g., for Change in Family Relations, in the questionnaire a rating of 5 denotes "much better" and 1 denotes "much worse. These values were subtracted from 6 to create the reversed score (1 denotes much better 5 denotes much worse).

For the Factor 2 score for Adolescents, loadings have been reversed here and in Figure 2 (left panel) to clarify that worsening of *Change in Family Relations* was correlated with worsening (positive change) of Mood8-Change scores, as shown in the Figure. This was true in Children as well.

Table S3: Diagnoses of adolescent cases

Diagnosis	Absent	Past only	Now only	Past & Now	ALL
Separation anxiety	103	9	0	11	20
Performance anxiety	90	9	0	24	33
Social Phobia	112	4	0	7	11
Other anxiety disorder	93	10	2	18	30
Obsessive-compulsive disorder	117	2	0	4	6
Any anxiety disorder	58	14	1	50	65
Somatization disorder	107	1	0	15	16
Adjustment disorder	105	4	1	13	18
PTSD	116	3	0	4	7
Attachment disorder	113	3	0	7	10
Acute psychotic episode	120	1	0	2	3
Schizophrenia	121	0	1	1	2
Depression	79	13	2	29	44
Bipolar disorder	119	2	0	2	4
Conduct disorder	106	7	0	10	17
Tourette’s disorder	121	0	0	2	2
Eating disorder	110	2	2	9	13
Other diagnosis	114	6	0	3	9
ADHD	99			24	
Intellectual deficiency	116			7	
ASD	113			10	
Learning disability	87			36	

Table S4: Diagnoses of child cases

Diagnosis	Absent	Past	Current	Past & Now	ALL
Separation anxiety	66	8	0	25	33
Performance anxiety	75	4	2	18	24
Social Phobia	90	3	1	5	9
Other anxiety disorder	83	0	2	14	16
Obsessive-compulsive disorder	98	0	0	1	1
Any anxiety disorder	46	7	4	42	53
Somatization disorder	98	0	0	1	1
Adjustment disorder	84	2	1	12	15
PTSD	97	1	1	0	2
Attachment disorder	88	4	0	7	11
Acute psychotic episode	97	2	0	0	2
Depression	89	2	1	7	10
Bipolar disorder	99	0	0	0	0
Conduct disorder	77	10	1	11	22
Tourette's disorder	96	0	0	3	3
Eating disorder	93	0	0	6	6
ADHD	65			34	
Intellectual deficiency	69			30	
ASD	78			21	
Learning disability	66			33	

Table S5: MOOD8 scores in relation to confinement and diagnosis (adolescents)

Diagnosis	Period	N	Mean	SD				t	df	P
**All** (during vs. before)	CONF1.	123	**19.69**	6.96				-3.67	122.00	**0.00036**
	Before		**21.89**	6.60						
		**Dx-**	**Mean**	**SD**	**Dx+**	**Mean**	**SD**	**t**	**df**	**P**
Any anx disorder	CONF1	58	**18.81**	5.96	65	**20.48**	7.71	-1.35	118.68	0.180
	Before		**21.02**	6.41		**22.68**	6.72	-1.40	120.46	0.164
	Change		**-2.21**	6.41		**-2.20**	6.91	-0.01	120.81	0.995
MDD	CONF1	79	**18.87**	6.33	44	**21.16**	7.83	-1.66	74.55	0.102
	Before		**20.03**	5.76		**25.25**	6.76	-4.33	77.78	**0.00004**
	Change		**-1.15**	6.02		**-4.09**	7.37	2.26	75.12	**0.027**
ADHD	CONF1	99	**19.75**	7.15	24	**19.46**	6.26	0.20	38.92	0.845
	Before		**22.30**	6.77		**20.21**	5.67	1.56	40.52	0.127
	Change		**-2.56**	7.06		**-0.75**	4.45	-1.57	54.91	0.123
ASD	CONF1	113	**19.99**	7.06	10	**16.30**	4.81	2.22	12.72	**0.045**
	Before		**21.93**	6.71		**21.50**	5.58	0.23	11.43	0.823
	Change		**-1.94**	6.65		**-5.20**	6.21	1.58	10.91	0.142
ID	CONF1	116	**19.72**	6.96	7	**19.14**	7.58	0.20	6.62	0.849
	Before		**22.09**	6.60		**18.57**	6.16	1.46	6.86	0.188
	Change		**-2.37**	6.70		**0.57**	5.56	-1.34	7.09	0.221

Shown are mean MOOD8 scores and SD before and during the first COVID-19 confinement. The ***first row*** shows Before and During scores for All participants and the results of a paired t-test demonstrating a significant change (lower MOOD8 during confinement). The ***remaining rows*** report data for participants without (Dx-) and with (Dx+) five frequent ICD-10 diagnoses and results of t-tests comparing those subgroups. The ***Change*** score is ***CONF1-Before***, so that a negative Change score reflects less low mood and worry during CONF1. Anxiety includes Separation Anxiety Disorder, Performance Anxiety Disorder, Social Phobia, Obsessive-Compulsive Disorder, or Other Anxiety Disorder. MDD = Major Depressive Disorder. ADHD = Attention Deficit Hyperactivity Disorder. ASD = Autism Spectrum Disorder (these were mostly higher-functioning ASD, without comorbid ID). ID = Intellectual Disability or Autism Spectrum Disorder. ID = intellectual Disability.

Table S6: MOOD8 scores in relation to confinement and diagnosis (children)

Diagnosis	Period	N Before	Mean	SD				T	df	P
All (during vs. before)	CONF1	99	18.43	6.18				-3.77	98.00	***0.00028***
	Before		20.77	5.68						
		**Dx-**	**Mean**	**SD**	**Dx+**	**Mean**	**SD**	**T**	**df**	**P**
Any anxiety disorder	CONF1	46	**17.50**	5.47	53	**19.25**	6.68	-1.43	96.70	0.156
	Before	46	**19.37**	4.55	53	**21.98**	6.30	-2.39	94.01	***0.019***
	Change	46	**-1.87**	5.25	53	**-2.74**	6.89	0.71	95.46	0.480
Conduct disorder	CONF1	77	**18.08**	6.03	22	**19.68**	6.67	-1.02	31.46	0.318
	Before	77	**20.39**	5.68	22	**22.09**	5.61	-1.25	34.33	0.219
	Change	77	**-2.31**	6.27	22	**-2.41**	5.92	0.07	35.64	0.947
ADHD	CONF1	64	**17.89**	6.30	35	**19.43**	5.90	-1.21	74.12	0.230
	Before	64	**20.47**	5.89	35	**21.31**	5.31	-0.73	76.47	0.469
	Change	64	**-2.58**	6.78	35	**-1.89**	4.90	-0.58	89.49	0.561
ID	CONF1	68	**18.40**	6.23	31	**18.52**	6.16	-0.89	58.72	0.930
	Before	68	**21.56**	5.81	31	**19.03**	5.04	2.20	66.45	***0.031***
	Change	68	**-3.16**	6.52	31	**-0.52**	4.93	-2.23	75.40	***0.029***

See legend for **Table S5**. ASD was not analyzed separately for children, because out of 22 ASD and 31 ID patients, 19 had both diagnoses.

Table S7: Lack of association of Mood8-Change with Sex or Age

Analysis	Subjects	N	t (df)
Mood8-Change X Sex (t-test)	Adolescents	119	0.388 (114.52)
	Children	98	0.360 (44.32)
			r
Mood8-Change X Age (Pearson r)	Adolescents	123	-0.005
	Children	99	-0.062

To determine whether either Sex or Age predicted Mood8-Change, in each cohort t-tests were carried out of Mood8-Change in males vs. females, and Pearson correlations were calculated between Mood8-Change and Age.

All p-values were > 0.10.

Note that for analyses of sex, Ns are reduced because there were 4 adolescent participants and 1 child participant with non-binary gender so that sex was reported as “other”..

Table S8: Linear mixed model analysis of Mood8 scores during CONF1 vs. the three months before CONF1, with adjustment for age and sex

(A) Adolescents

Effect	Beta	SE	t	p-value
**No covariates (N=123)**				
**Time**	-2.20	0.60	-3.672	***0.00036***
**Adjusting for Age (N=123)**				
**Time**	-2.20	0.60	-3.672	***0.00036***
**Age**	1.09	0.25	4.339	***0.00003***
**Adjusting) for Age and Sex (N=119)**				
**Time**	-2.48	0.59	-4.196	***0.000052***
**Age**	0.95	0.26	3.613	***0.00044***
**Sex**	-1.88	1.06	-1.770	0.079

(B) Children

Effect	Beta	SE	t	p-value
**No covariates (N=99)**				
**Time**	-2.33	0.62	-3.765	***0.00028***
**Adjusting for Age (N=99)**				
**Time**	-2.33	0.62	-3.765	***0.00028***
**Age**	0.03	0.17	0.184	0.85
**Adjusting for Age and Sex (N=98)**				
**Time**	-2.49	0.61	-4.110	***0.000082***
**Age**	0.01	0.17	0.032	0.97
**Sex**	0.57	1.14	0.504	0.62

These analyses utilized Linear Mixed Models to evaluate whether, in each cohort, there was a significant change in Mood8 scores during the first COVID-19 confinement period (CONF1, “lockdown”) compared with the scores for the three months prior to COVID. Each model included Subjects (random effect) and Time (fixed effect); subsequently Age and then Age and Sex were entered as fixed effects). Models were fitted by restricted maximum likelihood using R package *lme4*.

The analyses that included Sex as a covariate had reduced Ns because they omitted 4 adolescents and 1 child who were reported as “other” gender. This was because they were seen in a clinic for transgender issues. Thus we first entered Age alone to utilize the full cohorts, and then added Sex (coded categorically as “male” or “female”) as a second covariate after excluding those with “other” sex.

In all analyses, the Time effect remained robustly significant with negative t, ***demonstrating a reduction of Mood8 scores, on average, during the first lockdown period (CONF1).***

In Adolescents, older patients had higher Mood8 scores than younger patients across the two time periods. However, as shown in Table S7, there was no correlation between age and the ***change*** in Mood8 score.

Table S9: Change in clinicians' CGI-Severity ratings (5 time periods, Linear Mixed Model regression): all subjects, and subgrouped by presence of ID and/or ASD

A. Cohort/subgroup statistics; and overall significance of variance across time (LMM)

	**CGI_PAST**	**CGI_BEFORE**	**CGI_CONF1**	**CGI_DECONF**	**CGI_CONF2**	**Effect(s)**	**Chi-sq (df)**	**P-value**
**All cases N:**	138	139	138	134	128	Global	101.94 (9)	***6.38E-18***
						Time	42.99 (4)	***1.04E-8***
						Diagnosis	31.24 (1)	***2.27E-8***
						Time x Diagnosis	11.05(4)	***0.026***
**Arithmetic Mean**	3.21	3.08	2.92	2.75	2.88			
**Standard Error**	0.12	0.12	0.13	0.13	0.13			
**Standard Deviation**	1.42	1.47	1.51	1.484	1.51			
***With* ID and/or ASD N:**	35	35	34	34	32	Time	2.63 (4)	0.62
**Arithmetic Mean**	4.31	4.26	4.32	4.17	4.25			
**Standard Error**	0.20	0.21	0.23	0.24	0.24			
**Standard Deviation**	1.21	1.22	1.34	1.38	1.34			
***Without* ID or ASD N:**	103	104	104	100	96	Time	45.45 (4)	***3.20E-9***
**Arithmetic Mean**	2.84	2.68	2.46*	2.27*	2.43*			
**Standard Error**	0.13	0.13	0.12	0.12	0.13			
**Standard Deviation**	1.29	1.32	1.27	1.18	1.28			

Shown are the Mean, SE and SD of clinician CGI-S ratings for 5 time periods -- PAST (>3 months before COVID); the 3 months BEFORE confinement; CONF1 (first confinement); DECONF (first deconfinement); and CONF2 (second confinement) -- for the entire cohort (N=139), and for subgroups *with* Intellectual Deficiency and/or Autism Spectrum Disorder (N=35) or *without* either diagnosis (N=104), as illustrated in **Figure 4**.

The last 3 columns summarize the LMM analysis of CGI-S ratings using R package *lme4.* The model included Subjects (random effect, intercept); and Time, Diagnosis and Time x Diagnosis (fixed effects) (see Methods for details). Significance was evaluated by likelihood ratio tests. There was a significant interaction between time and diagnostic group (p=0,026): CGI-S differed across time points in those without ID or ASD (p=3.20E-9) but not in those with ID or ASD (p=0.62). For patients without ID or ASD, * indicates mean CGI-S values that were significantly different than the previous time period (see details in B below).

B. Pairwise comparisons of adjacent time periods (subgroup *without* ID or ASD, N=104)

**Time period comparison**	**beta**	**Se**	**t**	**P**
**BEFORE vs. PAST**	-0.129	0.069	-1.881	*0.060*
**CONF1 vs. BEFORE**	-0.146	0.069	-2.132	**0.033**
**DECONF vs. CONF1**	-0.154	0.069	-2.220	**0.026**
**CONF2 vs. DECONF**	0.149	0.071	2.103	**0.035**

As shown in (A) above, LMM analysis showed significant change of CGI-S score over time, but only in patients *without ID or* ASD. Here we show results of post hoc LMM analysis of differences in CGI-S scores between adjacent time periods. Consistent with the primary analyses of Mood8 scores in both Adolescents and Children, they show an ***improvement***, on average, during CONF1. This was followed by a modest ***additional improvement*** during DECONF and then a ***modest worsening*** during CONF2 (back to the CONF1 level).
